# The use of fluorescence enhancement to improve the microscopic diagnosis of falciparum malaria

**DOI:** 10.1186/1475-2875-6-89

**Published:** 2007-07-06

**Authors:** Rebecca Guy, Paul Liu, Peter Pennefather, Ian Crandall

**Affiliations:** 1 Department of Laboratory Medicine and Pathobiology, University of Toronto, Toronto, Ontario M5S 1A8, Canada; 2 Faculty of Pharmacy, University of Toronto, 144 College St., Toronto Ont., M5S 2S2, Canada; 3 Public Health Agency of Canada, Laboratory for Foodborne Zoonoses, Saint-Hyacinthe, Quebec, J2S 8E3, Canada

## Abstract

**Background:**

Giemsa staining of thick blood smears remains the "gold standard" for detecting malaria. However, this method is not very good for diagnosing low-level infections. A method for the simultaneous staining of *Plasmodium*-parasitized culture and blood smears for both bright field and fluorescence was developed and its ability to improve detection efficiency tested.

**Methods:**

A total of 22 nucleic acid-specific fluorescent dyes were tested for their ability to provide easily observable staining of *Plasmodium falciparum*-parasitized red blood cells following Giemsa staining.

**Results:**

Of the 14 dyes that demonstrated intense fluorescence staining, only SYBR Green 1, YOYO-1 and ethidum homodimer-2 could be detected using fluorescent microscopy, when cells were first stained with Giemsa. Giemsa staining was not effective when applied after the fluorescent dyes. SYBR Green 1 provided the best staining in the presence of Giemsa, as a very high percentage of the parasitized cells were simultaneously stained. When blood films were screened using fluorescence microscopy the parasites were more readily detectable due to the sharp contrast between the dark background and the specific, bright fluorescence produced by the parasites.

**Conclusion:**

The dual staining method reported here allows fluorescence staining, which enhances the reader's ability to detect parasites under low parasitaemia conditions, coupled with the ability to examine the same cell under bright field conditions to detect the characteristic morphology of *Plasmodium *species that is observed with Giemsa staining.

## Background

*Plasmodium falciparum *malaria is a medical emergency that requires correct diagnosis and appropriate treatment [1-4] The re-emergence of malaria in Africa and other regions is a disaster [5,6] and has lead to an increased number of imported cases in non-endemic regions. One diagnostic option is the use of rapid tests that detect *P. falciparum *histidine-rich protein 2 (HRP-2) in whole blood [7,8], however while these tests are available in some malaria-endemic regions, regulatory issues can limit their availability elsewhere. PCR methods are also effective for diagnosing malaria [9,10], however, they have not been adopted for widespread clinical use for regulatory and cost reasons [7,11]. The current "gold standard" for malaria diagnosis in most clinical laboratories remains microscopic examination of Giemsa-stained thick and thin blood films [12], but this method requires a reader with experience and well-developed pattern recognition skills to provide an accurate diagnosis [13-15]. Adding fluorescent dyes to blood samples to highlight the presence of parasites within erythrocytes has been considered as a potential method of improving the accuracy of microscopic diagnosis [16-18], but this strategy by itself has several limitations, including cost and regulatory issues. A method that would allow malaria parasites to be simultaneously stained with both a fluorescent nuclear stain, thereby allowing rapid screening of erythrocytes for parasitized forms, and with Giemsa stain, thereby permitting confirmation and speciation of the specimen to take place in the traditional manner, might improve reader performance.

The purpose of this study was to determine if a dual staining method combining protocols that are currently in use with a fluorescent stain of nuclear material in parasites could be developed with an ultimate goal of improving reader accuracy. Despite the development of new diagnostic technologies, microscopic examination remains the method commonly used to diagnose malaria [19] and therefore we attempted to develop an enhancement, rather than a replacement, of this method. A potential source of variation among the accuracy of readers may result from differing degrees of pattern recognition proficiency [15]. Blood films present the reader with potentially complex visual fields and frequently require accurate recognition of small numbers of parasites of variable morphology [7].

The use of fluorescence stains was designed to reduce the visual task of perceiving the presence of nucleated material contained in parasitized red blood cells (pRBCs), such that potential parasites could then be examined more carefully in the familiar Giemsa-stained format. Fluorescent dyes have been used in the analysis of malaria in a variety of ways including: rapid diagnostics [20], the detection of different stages of *Plasmodium *in culture, animal models using microscopy [21], flow cytometric determination of parasitaemia [22,23], for rapidly testing the efficacy of new therapeutics by use of nucleic acid staining of PicoGreen, and for rapid drug screening in microfluorimetric assays [24]. A combination of the fluorescent dyes DAPI and propidium iodide has previously been shown to markedly reduce the length of time required for detection of malaria parasites in thin blood smears when compared with Giemsa staining [20]. However, staining nucleic acids with fluorescent dyes alone is non-specific for *Plasmodium *and cannot be used to differentiate species of malaria.

Twenty two nucleic acid-binding fluorescence dyes were examined to determine how suitable they were for detecting *P. falciparum *within red blood cells using three commonly available excitation and emission filter sets. Dyes that provided clear and intense staining of nuclear material were then tested to determine if they continued to function in the presence of Giemsa stain. A combination of Giemsa followed by SYBR Green 1 was found to produce stained structures under light microscopy that permitted confirmation and speciation while still permitting screening for parasites under fluorescent microscopy. *P. falciparum *was used because it could be easily cultured, however since the other species of *Plasmodium *that cause human malaria can be visualized with Giemsa stain and contain nucleic acids the method should be applicable to them as well.

## Methods

### *Plasmodium falciparum *cultures

Parasites were grown in type O-positive blood obtained by venipuncture of volunteers. Cultures were maintained by the method of Trager and Jensen [25] by using RPMI 1640 supplemented with 10% human serum (a kind gift obtained under ethical consent from Chemo Day Care at the Princess Margaret Hospital, Toronto, Canada) and 50 μM hypoxanthine (Gibco, Grand Island, NY). Culture isolates of *P. falciparum *that were used included ITG, 3D7, FCR3 and two patient isolates. No difference was observed in the staining between these lines.

### Molecular dyes

The bright field microscopy stain evaluated was Accustain^® ^Giemsa Stain, Modified (SIGMA-Aldrich, St. Louis, Mo). The fluorescent dyes GelRed (Biotium Inc., Hayward, CA) and acridine orange (3, 6,-bis [dimethyl-amino] acridine hydrochlorate (Sigma, St Louis, MO) were suspended in distilled H_2_0 to a concentration of 10 mg/ml. Molecular Probes Inc. (Eugene, OR) was the source of SYBR Green I (supplied as a 10,000× concentrate in dimethylsulfoxide, DMSO); SYTO Red Fluorescent nucleic acid stains (SYTO dyes 17, 59, 60, 61, 62, 63, 64) (5 mM concentrations in DMSO); SYTO Blue Fluorescent Nucleic Acid Stains (SYTO dyes 40, 41, 42, 43, 44, 45) (5 mM concentrations in DMSO); YOYO-1 (1 mM in DMSO); LIVE/DEAD Reduced Biohazard Viability/Cytotoxicity Kit#1 component A (SYTO 10) (in DMSO) and component B (ethidium homodimer-2, EthD-2) (in 20% DMSO); 4',6-diamidno-2-phenylindole, dihydrochloride (DAPI) (powder made up in dimethylformamide (DMF) to a concentration of 1 mg/ml); Hoechst 33258 pentahydrate (bis-benzimide) (powder was suspended in DMF to a concentration of 0.67 mg/ml); and ethidium monoazide (EMA) (powder and suspended in sterile distilled H_2_0 to a concentration of 1 mg/ml).

### Fluorescent staining

Ten microliter samples of *P. falciparum *cultures (5% haematocrit with parasitaemia ranging from 1 to 5%) were prepared as films on glass slides. The films were air dried, fixed by flooding the slide with room temperature absolute methanol (MeOH) and allowed to air dry. Individual fluorescent stains were performed by adding 300 μL of the dye solution (diluted 1:500 in 10 mM Tris pH 8) directly to the dried film. The slide was then placed in the dark for 20 min at room temperature. Dye solution was then gently rinsed away using tap water and the slides were air-dried in the dark. A drop of emersion oil was then placed directly onto the slide and the blood film was examined using fluorescent microscopy. EMA photoactivation was performed by adding a 1:500 dilution of EMA in 10 mM Tris pH 8 to a culture film that had previously been hydrated with 10 mM Tris pH 8. A cover slip was then placed on top of the slide to prevent the EMA solution from drying and the slide was placed in the dark for 5 min to allow the dye to permeate the blood film. The slide was then placed 8 cm away a Super-Bright 20-LED Pivot Lantern (Innovage Inc., Outdoor™, Concord, ON), and exposed for 20 minutes. Residual EMA solution was gently rinsed off using tap water.

### Giemsa staining plus SYBR

Dual staining of culture films was carried out using a 1:25 dilution of Giemsa (Accustain^® ^Giemsa Stain, SIGMA) in 10 mM Tris pH 8 that had been passed through a 0.2 μm filter to remove undissolved material. Prior to use the diluted Giemsa solution was centrifuged at 10,000 × *g *for 2 min to pellet particulates in the solution. Giemsa stain (250 uL) was added to each slide by carefully spreading the solution across the entire blood film and left for 30 min at room temperature after which the stain solution was removed by rinsing with tap water. Excess water on the slide was removed and 300 μL of 10 mM Tris pH 8 was added. The Tris buffer was then removed and 300 μL of a 1:3,000 dilution of SYBR Green 1 in 10 mM Tris pH 8 was added. Care was taken to prevent the slide from drying prior to the addition of the SYBR solution to maximize SYBR Green staining. The slide was placed in the dark and left for 15 min at room temperature. The SYBR Green 1 solution was removed from the slide using a gentle stream of tap water prior to air drying in the dark and examination.

### Microscopy

Blood films were examined using a Nikon Labophot compound fluorescent microscope under 1000× magnification. Filter sets included: 1) Ex. 340–380, BA 435–485; 2) Ex. 450–490; 3). Ex. 546/10, BA 590 In some cases a custom built illuminator was used that employed a 5 mW Luxeon LED (Philips Lumileds Lighting Company, San Jose, CA) as the light source.

## Results and discussion

### Staining of *Plasmodium falciparum *cultures with DNA specific fluorescent dyes

A series of fluorescent dyes were tested for their ability to provide nucleic acid-specific fluorescence staining of malaria parasites within a red cell and that could easily be visualized using one of three standard filter sets: Ex. 340–380, BA 435–485 ("Fluorescein"); Ex 450–490/Em 520 ("DAPI"); and Ex 546/Em 590 ("rhodamine"). Several dyes produced intense fluorescence that was easily visible (Table 1 and Table 2). The optimal concentration of these dyes under the conditions employed was found to be a 1:500 dilution, with the exception of SYBR Green 1, which produced strong fluorescence at a 1:10,000 dilution.

**Table 1:** Fluorescence staining of pRBCs with different fluorescent dyes with differing absorption and emission spectra

Fluorescent dye	Spectra Em/Ex (nm)	Detection filter*	Intensity of fluorescent staining^†^
SYBR Green 1	497/520	2	++++
Ethidium monoazide (EMA)	504/600	3	
non photoactivated			+++
photoactivated			++++
Yoyo-1	491/509	2	+++
Acridine Orange	502/526		
Live/Dead Reduced Biohazard			
Viablity/Cytotoxicity Kit			
Component A (SYTO 10)	484/505	2	++
Component B (Ethidium homodimer-2 [EthD-2])	520/610	3	++++
A+B		3	++
blue fluorescent dyes			
SYTO 40	420/441	2	+
SYTO 41	430/454	2	+
SYTO 42	433/460	2	+
SYTO 43	436/467	2	+
SYTO 44	446/471	2	++++
SYTO 45	455/484	2	+++
red fluorescent dyes			
SYTO 17	621/634	3	+++++
SYTO 59	622/645	3	+++++
SYTO 60	652/678	3	+
SYTO 61	628/645	3	++++
SYTO 62	652/676	3	-
SYTO 63	657/673	3	+
SYTO 64	599/619	3	+++++
GelRed	500/600	3	++++
Hoescht 33258	345/487	1	++
DAPI	358/461	1	++

*Fluorescence filters: 1) Ex. 340–380, BA 435–485; 2) Ex 450–490/Em 520; 3) Ex 546/Em 590

^†^Intensity of fluorescence ranged from no parasite specific fluorescence (-), low (+) to very high (+++++).

**Table 2:** Comparison of fluorescent dyes for dual staining of pRBCs in combination with Giemsa staining.

Fluorescent dye	Detection filter	Giemsa and fluorescent dye (light/fluorescence)*
SYBR Green 1	fluorescein	+/+
Ethidium monoazide (EMA)	rhodamine	
non photoactivated		+/-
photoactivated		+/-
Yoyo-1	fluorescein	+/+
Acridine Orange	rhodamine	+/-
Ethidium homodimer-2 [EthD-2])	"	+/+
SYTO 44	fluorescein	+/-
SYTO 45	"	+/-
SYTO 17	rhodamine	+/-
SYTO 59	"	+/-
SYTO 61	"	+/-
SYTO 64	"	+/-
GelRed	"	+/-
Hoescht 33258	DAPI	+/-
DAPI	"	+/-

*Plus/minus detection of Giemsa or fluorescence

### Dual staining of parasitized red blood cells (pRBCs)

Dyes that demonstrated good nucleic acid-specific fluorescence (Table 1) were tested in combination with Giemsa stain. No fluorescence was observed with any of the dyes when simultaneous staining of Giemsa and fluorescent dye was performed, or when fluorescence staining preceded Giemsa staining. However, dual staining was observed with SYBR Green 1, YOYO-1 and Ethd-2 dyes when the blood films were first stained with Giemsa, followed by staining with the fluorescent dye (Table 2). Four factors were found to be important for optimal dual staining: 1) the blood films had to be stained with Giemsa first, followed by the fluorescent dye; 2) particulates within the Giemsa stain had to be removed from solution by centrifugation at 10,000 × g for 2 min; 3) a dilution of 1:25 of the Accustain^® ^Giemsa Stain, (SIGMA-Aldrich) was optimal for staining of culture films; however most importantly, 4) the culture film had to be hydrated prior to fluorescent staining as no fluorescent staining was observed if the cell film was allowed to dry prior to addition of SYBR Green 1.

Sequential staining was observed to reduce the intensity of the Giemsa stain. Several methods were tested to see if it was possible to enhance the Giemsa stain while maintaining the fluorescent staining. The first approach involved the use of different buffers, such as 10 mM Tris pH 7.2, or 30 mM potassium citrate in 10 mM Tris, pH 7.4. However, no significant enhancement in either Giemsa or fluorescence staining was observed with these buffers compared to the results obtained with 10 mM Tris pH 8.0.

Previous reports have demonstrated that the binding of fluorescent and other dyes is dependent on the ionic strength of the buffer [18,24] and nucleic acid binding can be shifted from intercalation to minor groove binding by increasing the ionic strength of the buffer. To determine if competition between visible and fluorescent chromophores could be reduced dual staining was conducted in the presence of four concentrations of NaCl (diluted in 10 mM Tris pH 8). The three ionic strengths were: 0 mM (control); 10 mM (low); 30 mM (medium); and 100 mM (high). The quality of staining with Giemsa alone in the presence of NaCl was not affected at lower concentrations; however the highest ionic strength resulted in poor Giemsa staining. "Ring" and "mature" stage parasites were pale blue and the blue staining appeared diffuse as opposed to the more solid colour observed at low ionic strength.

**Figure 1:**
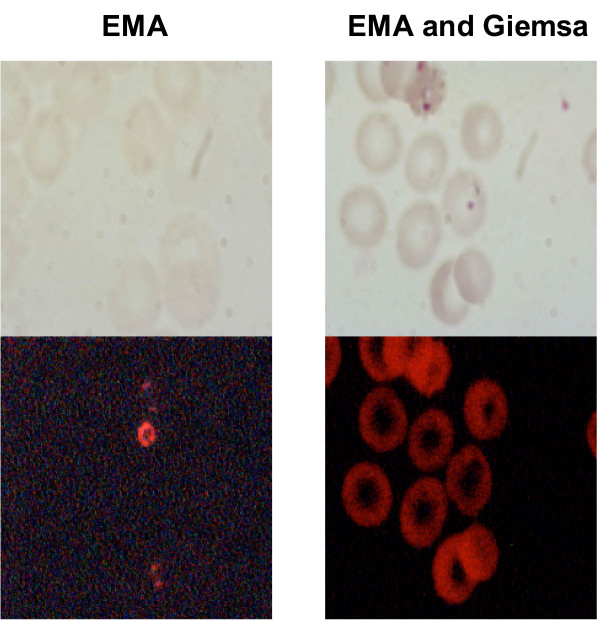
**Effect of EMA**. *P. falciparum*-parasitized red blood cells were stained with photoactivated ethidium monoazide (EMA) alone or with photoactivated EMA followed by Giemsa. The images on the top are viewed under light microscopy and the lower images are the same field viewed using fluorescent microscopy.

To determine if prior covalent attachment of a fluor produced acceptable results EMA was applied to the sample, photoactivated, unbound dye was rinsed away, and the sample was Giemsa stained. EMA becomes covalently linked to DNA upon photoactivation [26], therefore, covalently linked EMA might remain stably attached to DNA while it was stained with Giemsa and the intensity of the Giemsa stain might not be diminished due to leaching of the chromophores. Staining with photoactivated EMA followed by Giemsa resulted in superior Giemsa staining of pRBCs with well defined red and blue structures, however, a bright red uniform fluorescence of all RBCs was observed with no enhanced fluorescence of *P. falciparum*. To overcome the issue of non-specific fluorescent staining two washes with 40% MeOH (v/v) were used to remove excess EMA prior to Giemsa staining. While this step resulted in a very low level of uniform fluorescence of RBCs and bright parasite specific fluorescence prior to Giemsa staining, no parasite-specific fluorescence was observed following Giemsa staining (Figure 1). It remains to be determined whether the Giemsa staining is displacing the fluorescent dye or quenching EMA fluorescence.

### Dual staining with Giemsa and SYBR Green 1

The best combination of dyes for dual staining was observed to be Giemsa and SYBR Green 1. Fluorescent staining of *P. falciparum *cultures with SYBR Green 1 was well defined and rings appeared as small oval bodies within the red blood cells. This pattern was easily distinguishable from low intensity debris as it was uniform in shape and did not quench under exposure to excitation light. Non-parasitized red blood cells (RBCs) showed a low level of green fluorescence which made them easily visible, but they remained easily distinguished from pRBCs which contained brightly stained parasites.

**Figure 2:**
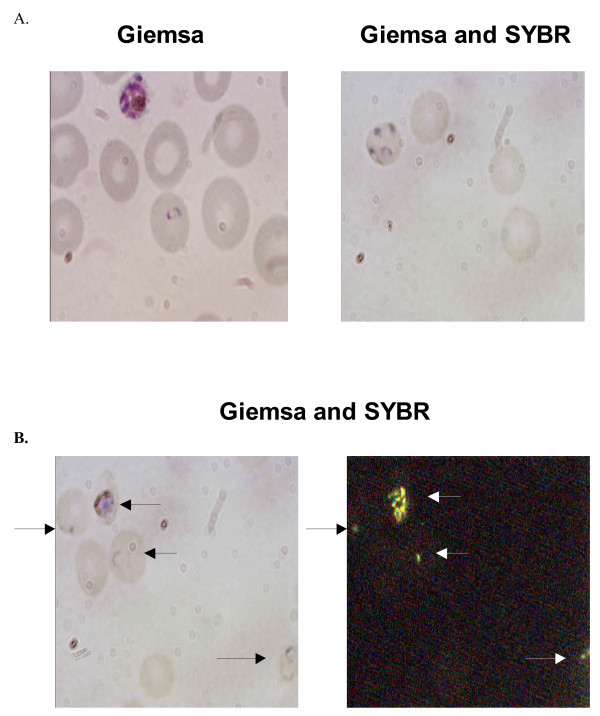
**Staining of *P. falciparum*-infected red blood cells**. Panel A depicts two different fields of stained *P. falciparum*-parasitized red blood cells, both viewed using light microscopy. The left hand side was stained with Giemsa alone and the right hand side was stained with Giemsa followed by SYBR Green 1. Panel B depicts the same field of dual stained *P. falciparum *infected red blood cells viewed under light (left hand side) or fluorescence (right hand side).

Cells stained with Giemsa alone showed the typical red staining of nuclear material and blue cytoplasmic staining (Figure 2A). However the intensity of Giemsa staining of *P. falciparum *cultures when stained with SYBR Green 1 was lower than with Giemsa alone (Figure 2B). The morphology of rings was not as well defined and frequently only blue staining was observed. Nevertheless mature parasites were easily visualized under light microscopy despite being paler and more diffuse.

A method that incorporates Giemsa staining with fluorescence called "Giemsa plus fluorescence" has long been in use for detection of chromosomes whereby the photosensitized nucleic acid binding fluorescent dye produces differential Giemsa staining as viewed under light microscopy [27]. However, this method does not appear to be directly applicable to malaria diagnosis due to the conditions used which do not allow visualization of the fluorescent dye but rather are to enhance visualization of structures under bright field.

It is possible that Giemsa can compete with other nucleic acids stains and displace them. Numerous fluorescent dyes have been developed that bind to a variety of molecules such as DNA, RNA, primary amines. While dual staining with two nucleic acid dyes was permissible using Giemsa followed by SYBR the staining was not optimal and one dye frequently competitively inhibited the other. For example, higher concentrations of Giemsa resulted in exclusion of SYBR. At lower concentrations of Giemsa, SYBR often displaced the red nuclear staining typically observed with Giemsa. Methylene Blue, a component of Giemsa, uses different mechanisms to bind to AT and GC bases of DNA. GC binding involves intercalation and optimal binding occurs at low ionic strengths [28]. AT coupling, on the other hand, occurs through minor groove binding and optimal binding is under high ionic strength. *P. falciparum *has a high AT content with an average of 80% for the entire genome and 90% in intergenic spaces and in introns [29]. SYBR Green 1 also binds through intercalation to DNA and is influenced by ionic strength [30], but attempts to shift either Giemsa or the fluor staining from intercalation to minor groove binding through increasing ionic strength was not effective in enhancing the Giemsa staining while maintaining good SYBR Green 1 binding.

### The application of dual staining, Giemsa + SYBR, to clinical blood films

**Figure 3:**
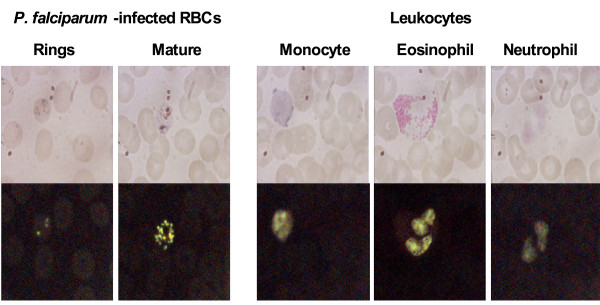
**Dual Giemsa and SYBR Green 1 staining of *P. falciparum *inoculated blood**. The top panel represents blood films visualized under light microscopy and the lower panel represents the same field visualized using fluorescence microscopy.

To determine if results obtained with pRBCs obtained from *P. falciparum *cultures were applicable to clinical samples, blood films (thick and thin) were prepared from whole human blood that either had low levels of *P. falciparum *added, or were read as positive by an experienced diagnostician. As was observed previously, there was little non-specific staining observed in the negative samples and the fluorescent staining that was present was easily distinguished from the characteristic pRBC pattern (Figure 3). The nuclei of leukocytes within the blood were also dual stained and provided a reference for the efficiency of staining for each blood film. A count of dual staining in 600 *P. falciparum *pRBCs revealed 100% dual staining with Giemsa and SYBR Green 1.

### LED illumination and computer based detection of pRBCs

**Figure 4:**
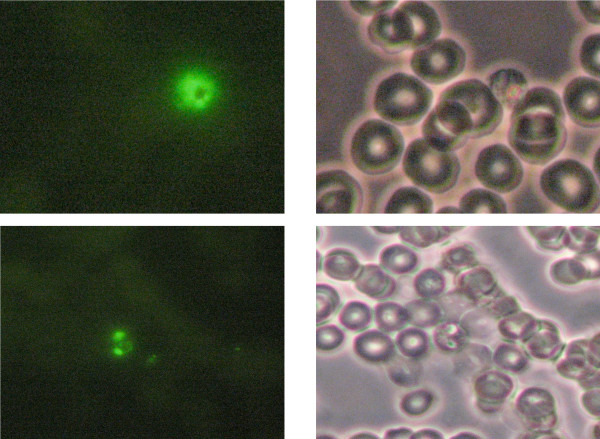
**LEDs can be used for detecting SYBR stained pRBCs**. Fluorescence emission from pRBCs excited with a 5 mW Blue LED powered by 4 AA batteries. The pRBCs were stained with SYBR Green 1 and were then examined using a Zeiss Axiostar Plus microscope with an LED light source fitted in place of its mercury lamp. Photographs depict fluorescent (left) and white light (right) images of a mature parasite (upper) and a ring stage parasite (lower). Fluorescent intensity of the stained parasites was comparable to that observed with the HBO 50/AC high intensity light source supplied by the manufacturer.

In order to determine if this method was compatible with an LED light source, a Luxeon 5 mW blue LED was mounted in place of the high intensity lamp used to excite fluors and used to excite a blood smear stained with both SYBR Green 1 and Giemsa. A white LED was used for bright field illumination. As seen in Figure 4 the quality of the stain was comparable to the use of incandescent and arc lamps of traditional microscopes. The use of LED light sources has several desirable features over conventional high intensity lamps as LEDs are inexpensive, light weight, can be powered for long periods by flashlight batteries, are physically robust, have a long service life, can be rapidly wand generate relatively little heat. These features allow their use in virtually any environment.

## Conclusion

Sequential Giemsa plus SYBR Green 1 staining of pRBCs as described herein provides a method of rapid screening and detection of the pathogen because the bright fluorescence of nuclear staining with SYBR Green 1 is well contrasted with the low RBC autofluorescence and the dark background. The combination of SYBR Green 1 for enhanced detection plus Giemsa for traditional identification and speciation appears to offer a superior diagnostic test.

In the same way that Giemsa is composed of a combination of dyes, it may be useful to combine different fluorescent dyes to further enhance pattern recognition and speciation (Figure 4). While nucleic acid specific dyes are frequently used to stain fixed tissue, numerous other dyes are known to interact with malaria parasites [31], therefore, future avenues of research may lead to vital staining of live parasites in RBCs. The emergence of virtual slide technology for tele-pathology applications [20] suggests that entire blood films can be digitally scanned in a sequential manner for sensitivity to different dyes and then the gold standard Giemsa stain can be applied at the end to provide a reference link to prior observations. The availability of vital dyes, some of which are substrates for the same transporters whose enhanced activity are responsible for resistance to anti-malarial drugs in certain strains of malaria [32], means that these dyes might also be used to characterize responsiveness and resistance to drug therapy.

At first glance, due to the high capital cost and high running costs of mercury lamps fluorescent-based tests would appear to be impractical for routine use in many clinical laboratories in the developed world and out of reach for laboratories in the developing world. However, the recent emergence of LED based light sources to replace arc lamp and laser sources [33,34], promises to simplify the use of routine fluorescence screening and reduce costs.

Further, the increased contrast provided by fluorescence based localization of pRBCs should facilitate machine vision approaches for automated screening of blood smears for parasitaemia levels and for speciation. This in turn sets the stage for remote tele-cytometry [35] of the samples which may address the experience deficit in dealing with malaria samples. It may, therefore, be practical to introduce an enhanced staining method that will reduce the requirement for high levels of skill and experience presently required to accurately diagnose malaria and increase the reliability of these tests.

## Authors' contributions

RG and PL performed the cell staining and evaluation, PP and IC conceived of the study, with PP providing expertise on microscopy and staining methods and IC providing malaria culturing and diagnostic guidance. All authors contributed to the preparation of the manuscript and have read and approved of all submitted versions, including its final form.
